# Hallmarks of exosomes

**DOI:** 10.2144/fsoa-2021-0102

**Published:** 2021-11-05

**Authors:** Nihat Dilsiz

**Affiliations:** 1 Molecular Biology & Genetics, Faculty of Engineering & Natural Sciences, Istanbul Medeniyet University, Istanbul, 34700, Turkey

**Keywords:** biomarkers, body fluids, exosomes, hallmarks, miRNAs

## Abstract

Exosomes are a new horizon in modern therapy, presenting exciting new opportunities for advanced drug delivery and targeted release. Exosomes are small extracellular vesicles with a size range of 30–100 nm, secreted by all cell types in the human body and carrying a unique collection of DNA fragments, RNA species, lipids, protein biomarkers, transcription factors and metabolites. miRNAs are one of the most common RNA species in exosomes, and they play a role in a variety of biological processes including exocytosis, hematopoiesis and angiogenesis, as well as cellular communication via exosomes. Exosomes can act as cargo to transport this information from donor cells to near and long-distance target cells, participating in the reprogramming of recipient cells.

## Lay abstract

Exosomes have been found in all of the body fluids, and the content of exosomes’ complex cargo is easily accessible by biological fluid sampling. The potential utility of exosome-based liquid biopsy in the diagnosis and prognosis of cancer and some other disorders has been highlighted. Exosomes can be engineered to transport a variety of therapeutic payloads, such as antisense oligonucleotides, immunological modulators, therapeutic drugs and vaccines, and they can be directed to a specific target. For the first time, ten different exosome hallmarks have been categorized according to their functional capabilities in the present review article.

Almost every cell in our body releases extracellular vesicles (EVs), which are nano-sized lipid bilayer membrane vesicles that are released into the extracellular environment. These EVs are currently classified as exosomes, microvesicles and apoptotic bodies based on their size, biogenesis, molecular composition and functions. They have been detected in a variety of bodily fluids, including circulating blood, breast milk, bile, saliva, nasal secretion, semen, cerebral spinal fluid, lymph, tear, urine and amniotic fluid (Figure 1).

**Figure 1.**
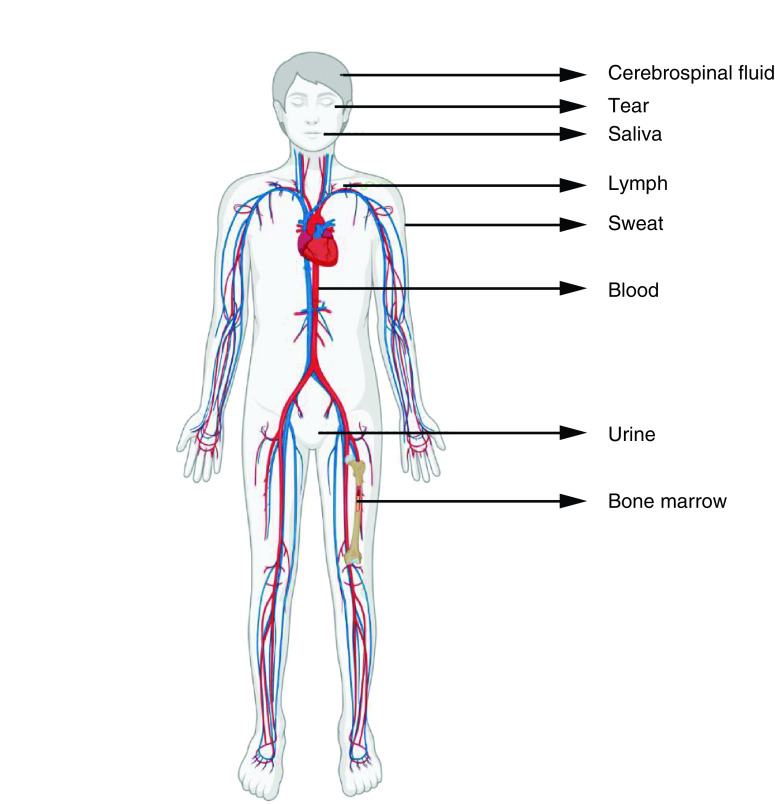
The various biological fluids in the human body.

Exosomes are the smallest EVs in terms of size, have a cup-shaped morphology when viewed under cryoelectron microscopy and are more homogenous in shape when compared with other EVs (Figure 2). Exosomes were discovered initially by Pan and Johnstone in 1983 during the process of erythrocytes maturation from sheep reticulocytes [1]. Exosomes are biological nanoparticles formed by almost all normal and diseased cell types and are found in all body fluids as well as *in vitro* grown cell lines with an average diameter of between 30 and 100 nm in size [2].

**Figure 2.**
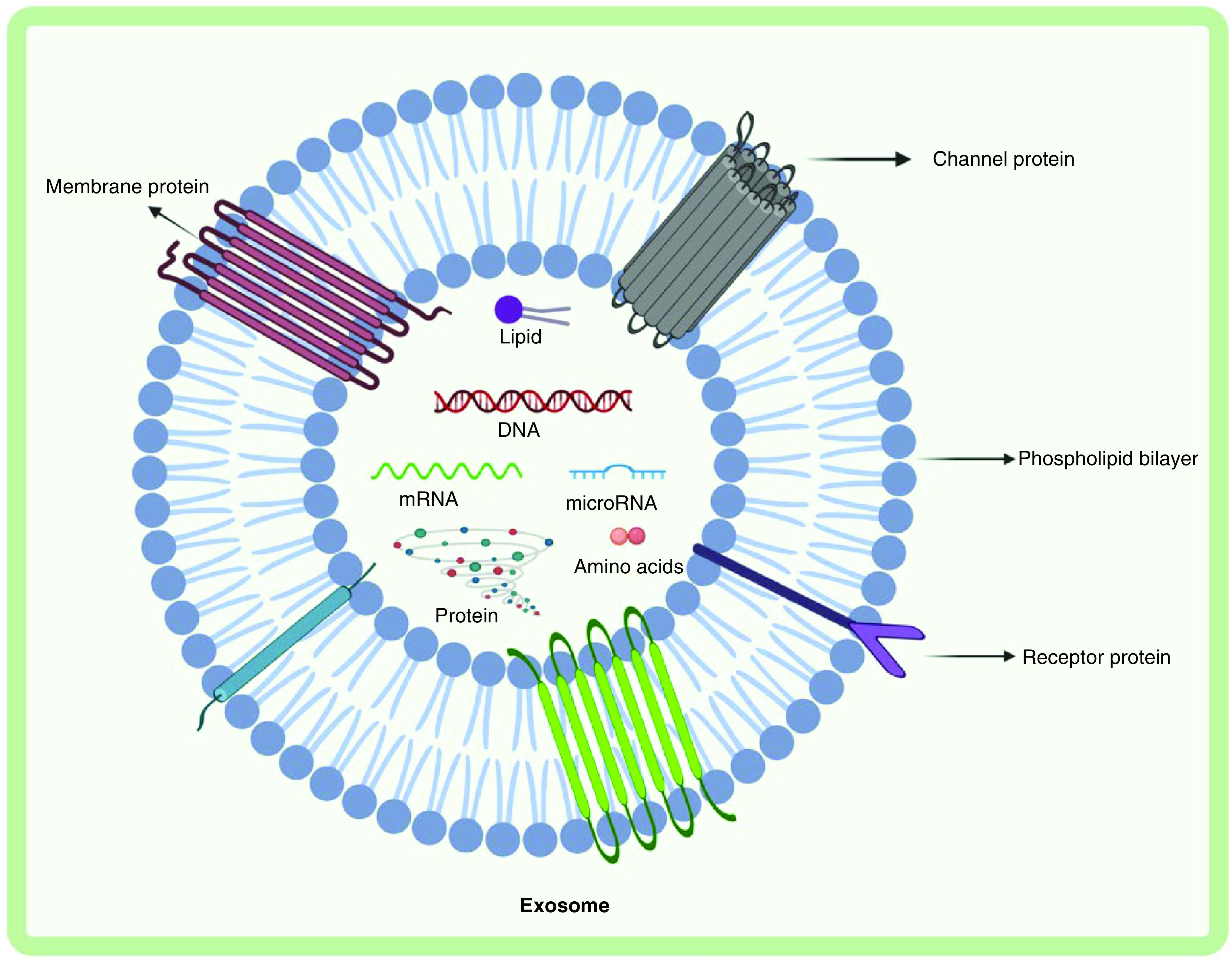
The exosome's structure. Exosomes contain DNA, RNA, proteins, lipids and metabolites. Exosomes are composed of various proteins: transmembrane proteins such as tetraspanins, antigen presenting molecules, glycoproteins and adhesion molecules; proteins in exosome lumen such as heat shock proteins (Hsp), cytoskeletal proteins, ESCRT components, membrane transport, fusion proteins, growth factors and cytokines. Exosomes also comprise of multiple lipids such as cholesterol, ceramides, sphingomyelin, phosphatidylinostol (PI), phosphatidylserine (PS), phosphatidylcholine (PC), phosphatidylethanolamine (PE) and gangliosides (GM) along with nucleic acids such as mRNA, miRNA, non-coding RNA and DNA in their lumen. ESCRT: Endosomal sorting complex required for transport; FasL: Fas ligand; Hsc: Heat shock cognate; TfR: Transferrin receptor; TGF: Transforming growth factor; TNF: Tumor necrosis factor; TRAIL: TNF-related apoptosis-inducing ligand; TSG: Tumor suspectibility gene.

Exosomes are formed by the endocytic pathway (endocytosis), which involves the inward budding of intraluminal vesicles from the plasma membrane, and are secreted and released into the extracellular space (exocytosis) after late endosomes or multivesicular bodies fusion with the plasma membrane (Figure 3) [3–9]. The multivesicular body can either be degraded by fusing to lysosomes or autophagosomes, or it can fuse to the plasma membrane and release exosomes [10,11]. The released exosomes might be absorbed by target cells through three possible pathways, namely, fusion with the cell membrane, endocytic absorption and ligand or receptor interaction [12].

**Figure 3.**
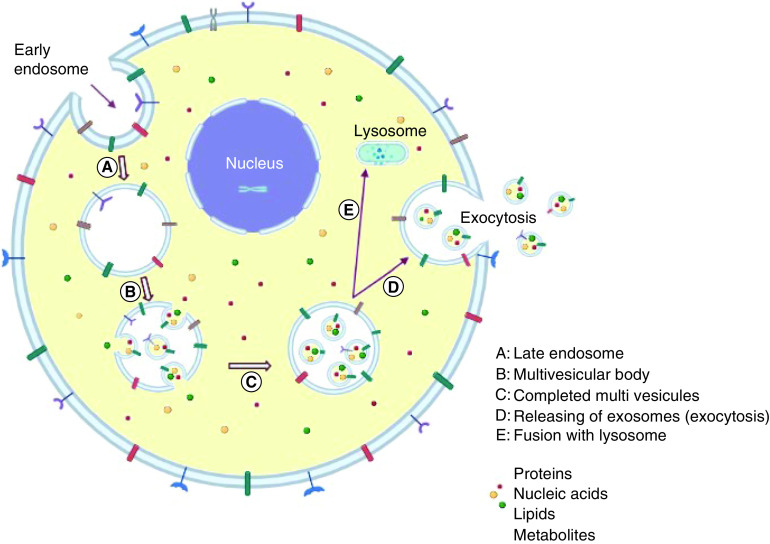
The biogenesis of exosomes and release pathway. **(A)** The exosome is derived from early plasma membrane-formed endosomes (endocytosis). **(B)** Further, early endosomes are transformed into late endosomes. **(C)** After that, it forms early multivesicular bodies. **(D)** Multivesicular bodies of the late stage. **(E)** In order to release exosomes, late multivesicular bodies may either be joined with autophagosomes and follow the degradation pathway through lysosomes or they can be fused to the plasma membrane via the microtubules and cytoskeletal network, and be released by budding from the cytoplasmic membrane (exocytosis).

Exosomes are a popular topic in medicine right now, with a lot of promise in terms of treating disease with agents derived from abnormal cells. Exosomal contents, including DNA fragments (both from the nucleus and mitochondria), various RNA species (coding and noncoding RNAs), cytosolic and cell surface proteins, and small metabolites, have been shown in many studies. Noncoding miRNAs are one of the most abundant cargos found in exosomes. These bioactive molecules are transferred from donor cells to target cells by exosome transport system, causing recipient cells to be reprogrammed. Exosomes can be purified from the fluids of a patient and transferred to the same patient with their new cargo for targeted tumor therapy after being modified [13–15]. The therapeutic cargos such as miRNAs or drugs can be loaded into native exosomes isolated and purified from cell medium and delivered by free diffusion, incubation, sonication, freeze–thaw cycles or electroporation [16].

It is vital to remember that exosome extraction methods are continuously improving, and that current biomarkers may only recognize a subset of exosomes with certain contents [10,17–20]. As a result of the adoption of new technologies, certain findings will need to be revised. Exosomes’ impact on recipient cells might vary due to their diverse expression of cell surface receptors, resulting in one set of exosomes driving cell survival, another set promoting cell death and yet another set stimulating immunomodulation, among other aspects, in various target cell types.

Exosomes have an impact on a broad range of physiological processes, including immunological responses [21,22], maintaining stem cells [23], tissue repair [24,25], CNS communication [26] and pathological processes in cardiovascular disorders [27,28], neurodegeneration [29], tumorigenesis [30] and inflammation [31]. Researchers are increasingly interested in the potential of these little wonder vesicles to help with gene transfer, disease diagnostics, cellular communication, drug delivery and biomarker-driven therapy [32]. The development of engineered exosomes is an ongoing research field that promotes the evaluation of diverse therapeutic cargoes, target selectivity enhancement, minimizing drug accumulation in nontarget tissue/organs and manufacturing optimization. Clinical investigations on their usage as biomarkers for diagnosis, disease severity and therapeutic response, as well as engineering applications as therapeutic cargo delivery vectors, are now being developed and rapidly translated for human applications. These approaches emphasize the importance of improved exosome-mediated cellular communication [33–35].

Recent data suggest that exosomal miRNAs are important players in the pathophysiology of many, if not all, human diseases including cancer, neurodegenerative and cardiovascular diseases, and wound healing [36]. miRNAs are mainly located in the cytosol but they are also packaged as cargo in exosomes where they are protected from degradation by RNase present in different biological fluids [37]. miRNAs packaged in exosomes act as post-transcriptional gene regulators by binding to target mRNAs, blocking mRNA translation or degrading the mRNA and therefore silencing the gene expression. Depending on the target mRNA, miRNAs can act as tumor suppressors or onco-miRNAs, and they play a key role in tumor growth, progression, therapy resistance and metastasis. In tumor cells, miRNA expression can be dysregulated, resulting in the overexpression of oncogenic miRNAs and the underexpression of tumor suppressor miRNAs. Many oncogenic miRNAs play a key role in cell proliferation, metastasis and the activation of oncogenic transcription factors, which have been shown to be overexpressed in various cancer cells.

They have enticing potential to be employed in therapeutic settings as an alternative to transcription factors during the reprogramming of a variety of somatic cells due to their small size, tissue-specific expression, capability of safe delivery into cells, regulation of a large number of genes, interlinked networks and pathways, and finally their involvement in cell fate transitions [38]. In the light of the information obtained to date, up to ten hallmarks of exosomes can be categorized according to their functions as follows (Figure 4):

- Regulation of gene expression
- Intracellular signaling and cell-to-cell communication
- Reprogramming of target cell
- Survival and proliferation
- Cellular differentiation and neoplasia
- Modulation of immunosystem
- Inducing angiogenesis
- Activating invasion and metastasis
- Diagnostic and prognostic biomarkers
- Delivery vehicle (drugs, vaccines, proteins and nucleic acids).

**Figure 4.**
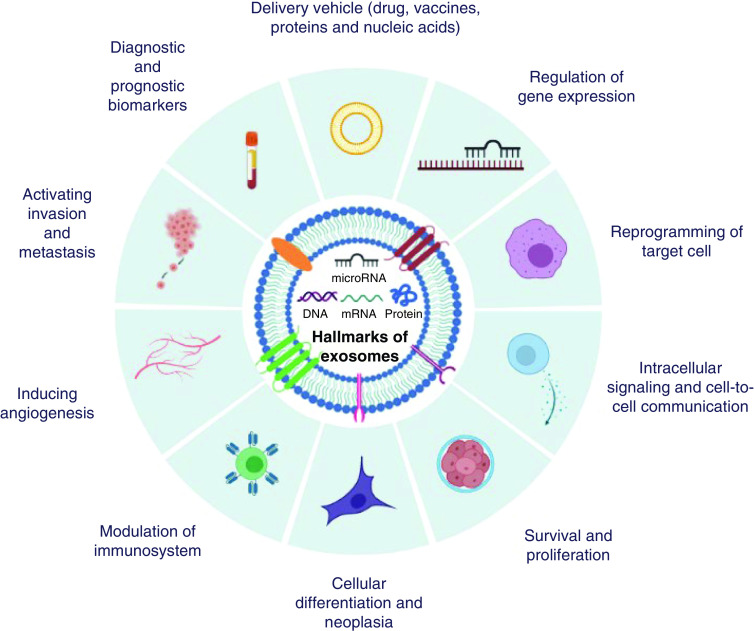
Hallmarks of exosomes.

## Regulation of gene expression

Endogenous miRNAs have been found to directly affect gene expression in the nucleus at the transcriptional level, in addition to mRNA post-transcriptional regulation in the cytoplasm, according to various studies. Exosomal miRNAs can impact the expression of epigenetic machinery components by targeting epigenetics-related enzymes like DNA methyltransferases (DNMTs), histone deacetylases, histone methyltransferases and ten–eleven translocation enzymes [39]. In the recent years, the impact of miRNAs on epigenetic expression, such as DNA methylation, DNA demethylation, RNA modification and histone modification, has become more widely acknowledged. miRNAs can impact the overall methylation profile of the genome by regulating the expression of DNA methylation-related enzymes.

The miR-29b was the first epigenetic miRNA to be discovered. It causes global DNA hypomethylation by suppressing DNMTs expression, either directly targeting the 3′-UTR regions of DNMT3A and DNMT3B or indirectly targeting specificity protein 1 (Sp1), a transactivator of the *DNMT1* gene in the case of DNMT1 [40–42]. In lung cancer, tumor-suppressor genes like fragile histidine triad enzyme and WW domain containing oxidoreductase that were epigenetically repressed by promoter hypermethylation were re-expressed as a result of promoter CpG island demethylation after miR-29b reintroduction [40,43]. miRNAs can also regulate histone modification in addition to DNA methyltransferases. Histone modifications such as H3K4me1, H3K27me3, H3K27ac, H3K9ac, H3K4me3 and H2AZ are found to be regulated by miRNAs during human spermatogenesis, according to bioinformatics analyses [39,44].

miRNAs also have a key role in regulating the cell cycle of embryonic stem cells (ESCs). miR-290-295, miR-302, miR-17-92, miR-106b-25, miR-106a-363 clusters and let-7 family are the most commonly expressed miRNAs in ESCs, accounting for roughly 70% of total miRNA molecules [45–48]. The G1/S transition is contributed by these miRNAs, which decrease the expression of retinoblastoma proteins in ESCs [46]. However, more research into these pathways, as well as the link between epigenetics and miRNAs, is required. This will help us to better understand how the human genome is controlled and also how mutagenic gene expression abnormalities might be treated therapeutically.

## Intracellular signaling & cell-to-cell communication

Exosomes transport encapsulated proteins and genetic information to destination cells, acting as information messengers from one cell to another. Exosomes have an effect on recipient cells through influencing a vast number of genes and intracellular signaling pathways, resulting in changes in host–cell function or phenotype. They mediate the transport of various signaling molecules from abnormal cells to normal cells, which can be negatively regulated. Exosomes play a key role in cell-to-cell communication between both nearby and distant tissue cells, causing physiological changes in recipient cells by transferring their cargo, and they have been linked to a number of pathologies including cancer, neurological disorders, cardiovascular diseases and autoimmune diseases [49,50]. Exosomes are of great interest to molecular biologists because they are produced by a unique intracellular regulatory process that defines their composition and, potentially, their functions once released into the extracellular environment [10,51–53]. However, improving our understanding of how exosomes provide their signaling function would require the development of improved tools to investigate the intracellular pathways of exosomes and their payloads.

## Reprogramming of target cell

Cell reprogramming is the process that transforms a differentiated somatic cell into a pluripotent stem cell (PSC) form or, in some cases, generating a new individual under certain circumstances. The reprogramming efficiency of induced PSCs (iPSCs) is regulated by miRNAs. Overexpression of the miR-290 or miR-302 families, for example, improves reprogramming efficiency [54]. Surprisingly, miRNAs have the ability to directly transform somatic cells into iPSCs. Human skin cancer cells, for example, have been demonstrated to be reprogrammed into pluripotent state using the miR-302 cluster [55]. It has also been shown that the direct transfection of mature miR-200c, miR-302 and miR-369 into mouse and human somatic cells can also transform them into pluripotent states [56]. In addition, miRNAs from the miR-30/let-7 family, which are downregulated, and those from the miR-17, miR-19, miR-290 and miR-8 families, which are upregulated, play essential roles in the activation and maintenance of pluripotency [57]. The three phases of the miRNA-mediated stem cell reprogramming process are initiation, maturation and stabilization [58]. miR-138 and miR-302/367, for example, promote iPSC generation while miR-34a and miR-195 inhibit it. In addition, miR-21, miR-1, miR-155, miR-184, miR-199a, miR-199b, miR-211 and miR-449 induce differentiation, while miR-495 inhibits it [59].

The tumor microenvironment (TME)’s reprogramming is critical for the development and establishment of the malignant phenotype [60]. Tumor-derived exosomes deliver tumor-promoting chemicals to recipient cells, causing abnormal signaling pathways to activate and mutations to occur, causing irreversible changes in the target cells. Additionally, a study looking at the role of exosomal miR-93 in hepatocellular carcinoma tumorigenesis found that it promotes proliferation and invasion, and that exosomal miR-93 overexpression predicts poor prognosis in hepatocellular carcinoma patients [60,61].

## Survival & proliferation

Human growth, development, reproduction and genetics are all based on cell proliferation, as we all know. Changes in the expression or activity of cell cycle-related proteins are also important in tumor development [62,63]. Exosomes produced from tumor cells have been found to stimulate tumor growth by directly activating signaling mechanisms such as phosphorylated phosphatidylinositol 3-kinase/protein kinase B (P13K/AKT) or mitogen-activated protein kinase/extracellular signal-regulated kinase (MAPK/ERK), which are responsible for sustaining the tumor proliferation [64–68]. Exosome-mediated activation viva P13K/AKT or MAPK/ERK pathways were found to induce cell proliferation in gastric cancer cells [66]. Another study of gastric cancers indicated that exosomal cluster of differentiation 97 protein (CD97) is involved in MAPK pathway-mediated proliferation [67]. Exosomes derived from bladder cancer and oral squamous carcinoma cells have also been shown that the activated PI3K/AKT and MAPK/ERK pathways stimulate cell proliferation [68,69]. It has been reported that exosomes generated from platelets transferred glycoprotein IIb–IIIa (CD41) to the surface of lung cancer cells by inducing the expression of G1/S-specific cyclinD2 in lung cancer cells aiming to promote the phosphorylation of MAPKp4244; also, the exosomes stimulated lung cancer cell proliferation [70].

Tumor cells-derived exosomes can modify the microenvironment, facilitating disease invasion and dissemination, in addition to their impact on cell proliferation. Exosomes derived from prostate cancer cells, in particular, have been found to transform fibroblasts into activated fibroblasts or myofibroblasts by transferring TGF-β to the extracellular medium [64,71,72]. Fibroblasts are abundant in tumor tissues, and their active form is well known for their function in tumor progression via growth factor release [51,73]. Similarly, exosome-mediated TGF-β transfer induced differentiation and activation of fibroblasts in bladder cancer [74].

## Cellular differentiation & neoplasia

Cell differentiation, neoplasia, tumor development and metastasis, paraneoplastic disorders and therapeutic resistance are all influenced by exosomes. The involvement of exosomes in the progression of cancer is believed to be dynamic and dependent on the type of cancer, genetics and stage of the disease [10]. Through the transfer of their miRNA cargo, exosomes derived from breast cancer and prostate cancer cells promote neoplasia. In exosomes from prostate cancer cells, miR-125b, miR-130 and miR-155, as well as Harvey-Ras and Kirsten-Ras mRNAs, play a key role in neoplastic reprogramming and tumor development of adipose stem cells [10,75,76].

### miRNAs & stem cell differentiation

Stem cells have distinct miRNA expression profiles that influence their fate [77]. This expression profiles could be used to terminally differentiate somatic cells from stem cells in order to treat a variety of diseases, such as myocardial infarction, neurological diseases, muscle diseases and blood diseases. On the one hand, miR-499, via targeting Sox6, enhances cardiovascular differentiation of human cardiomyocyte progenitor cells [78]; on the other hand, miRNAs regulate the cardiovascular differentiation of ESCs and iPSCs. Through the phosphatase and tensin homology deleted on chromosome ten (PTEN)/Akt pathway, miR-1 enhances cardiac differentiation of ESCs and suppresses cardiomyocyte apoptosis in the infarcted heart [79]. miR-1 also activates Kruppel-like factor 4 in retinoid acid-induced ESCs, which stimulates smooth muscle cells differentiation [80]. It has been reported that miR-199b regulates signal transducer and activator of transcription 3/VEGF signaling, which promotes EC differentiation in mouse iPSCs [81].

### miRNAs & neural differentiation

Previous researches have shown that miRNAs have also play a crucial role in neurogenesis. Doetsch and colleagues reported that miR-124 stimulates neuronal differentiation in the subventricular zone, which is the largest neurogenic niche in the adult mammalian brain, according to [82]. Forced expression of miR-34a reduces dendritic length, neuron branch counts and functional synapses, as well as disrupting inhibitory inputs [83]. More importantly, miRNAs regulate ESC and iPSC neurogenesis by targeting relative neuronal differentiation genes. In human iPSCs and ESCs, miR-371-3 is abundantly expressed. It has been reported that miR-371-3 suppression increases neuronal differentiation [84].

### miRNAs modulate osteogenic & chondrogenic differentiation of stem cells

The regeneration ability of osteogenic and chondrogenic cells from adult stem cells is extremely useful in medical research. miRNAs control osteogenic and chondrogenic differentiation throughout skeletal development by targeting key transcriptional factors and pathways. In osteoblast differentiation, the mitogen-activated protein kinase (MAPK also known as ERK)-dependent pathway is crucial. It promotes osterix expression and activates alkaline phosphatase by enhancing the phosphorylation of runt-associated transcription factor-2. By directly targeting focal adhesion kinase and downstream signaling, miR-138 suppresses the differentiation of human mesenchymal stem cells (MSCs) into osteoblasts [85]. miR-23b stimulates chondrogenic differentiation in human MSCs via inhibiting protein kinase A signaling [86]. It has been found that overexpression of miR-335-5p, which targets disheveled associated activator of morphogenesis 1 and rho-associated coiled-coil containing protein kinase 1, greatly increases chondrogenic differentiation of mice MSCs [87].

### miRNAs regulate hematopoietic differentiation of stem cells

miRNAs play a significant role in hematopoiesis in mammals. AAAGUGC seed-containing miRNAs promote primary hematopoietic progenitors when they are expressed ectopically [88]. B-lymphocyte differentiation is considerably aided by miR-181 [89].

## Modulation of immunosystem

Exosome-derived miRNAs act as a bridge between cancer cells and immune cells, allowing them to communicate with one another. Exosomes can both activate tumor development by generating antitumor immunological responses and repress tumor growth by evoking immunosuppression and evading immune surveillance in cancer. Exosomes’ immunostimulatory effects are mostly dependent on the antigen presentation they carry, whereas their immunosuppressive effects are primarily dependent on the ligands, proteins and miRNAs they contain, which inhibit antitumor cytotoxic T lymphocytes activity or increase immunosuppressive cells. Exosomes’ ability to express tumor-related antigens, MHCs and certain chemokines as well as induce helper T-cell immune responses, raises the possibility of using them as anticancer vaccines [90]. The ability of tumor cells to evade immune monitoring has been identified as a fundamental aspect of metastasis and has been linked to therapeutic resistance [60]. Exosomes have been linked to immune system dysfunction in both the innate and adaptive systems.

Tumor-derived exosomes from pancreatic cancer cells were found to reduce the expression of two key cytokines, tumor necrosis factor and interferon, in innate natural killer cells, as well as natural killer cell cytotoxicity against cancer stem cells, which are thought to be responsible in the metastatic process [60,91]. T-cell-mediated antitumor immunity can also be boosted by vaccination with tumor antigen-loaded dendritic cell-derived exosomes[12,64]. Indeed, many tumor cell-derived exosomes carry molecules from the parent tumor cells that might alter immune cell activation, cell development and anticancer actions directly or indirectly [64,92–94]. As a result, cancer antigens found in exosomes are being investigated as cancer vaccines in immunotherapy [95,96].

Exosomes influence immunosuppression, tumor growth and response to existing cancer therapies by modulating basic functional aspects of the lymphoid myeloid components of the TME [97]. Exosomes can be used therapeutically as a powerful weapon to activate antitumor immunity, which could lead to new cancer therapy options. Exosomes have been used in the development of therapies that target T lymphocyte and antigen-presenting cell-mediated immunity so far [97]. Despite the fact that this study has shown advanced and remarkable findings, the search for an effective cancer vaccine is still ongoing.

## Inducing angiogenesis

Angiogenesis (the generation of new blood vessels) is a natural process in which pre-existing vessels are used to create new blood vessels. It occurs often in organisms throughout their growth and development as well as in response to any injuries [64,98]. This process, however, is critical in the progression of cancer because tumor growth requires the rapid formation of vasculature to provide access to nutrition, oxygen and waste elimination [64]. Angiogenesis is essential for malignant tumor development and metastasis because new blood vessels provide more oxygen and nutrients while simultaneously removing waste products [99]. Angiogenesis is defined as the formation of novel thiny blood vessels from existing blood vessels, as well as complex endothelial cell activities, all of which are associated by increased vascular permeability, which promotes tumor growth and metastasis.

Exosomal miRNAs play a significant role in tumor-associated angiogenesis, according to new studies. Exosomes have a significant role in angiogenesis by transporting miRNA, mRNA and proteins, according to several studies [10,100,101]. For example, Umezu *et al.* found that exosomes derived from leukemia cells overexpress miR-92a (a miRNA that belongs to the mir-17–92 cluster) when they reach endothelial cells, resulting in increased motility and tube formation [100]. Exosomes can transport Delta-like 4, a membrane-bound Notch ligand that plays a key role in vascular formation and angiogenesis, through the 3D collagen matrix and to distant cells [101].

Exosome-induced uncontrolled cell proliferation results in a lack of oxygen and nutrient flow in the TME, causing hypoxia, that promotes epithelial-to-mesenchymal transition and a more invasive phenotype [60,102,103]. To maintain its microenvironment and metastasis, a developing tumor requires neovasculature for sufficient oxygen and nutrition supply [60,104]. Exosomes have also been linked to TME angiogenic and extracellular matrix remodeling, which is a crucial stage in tumor growth and metastatic dissemination. Such as endothelial tight junction zonular occludens 1 (ZO-1) expression is suppressed by breast cancer cell-derived exosomal miR-105, resulting in enhanced metastasis via decreasing blood vessel integrity and increasing vascular permeability [10,105].

Exosomal miR-155-5p derived from melanoma could induce fibroblasts to express proangiogenic factors such as VEGFa, FGF2 and others, giving novel strategies to suppress melanoma proliferation through increased cytokine signaling 1. Breast cancer cell-derived exosomal oncogenic miRNAs, such as miR-105 suppress endothelial tight junction ZO-1 expression, contributing to increased metastasis by impairing blood vessel integrity and enhancing vascular permeability, have been implicated in the angiogenic and extracellular matrix remodeling of the TME [10,106].

miRNAs found in the exosome, such as miR-9, miR-21, miR-23a, miR-29b, miR-92a, miR-103, miR-105, miR-126, miR-132 miR-135b, miR-155, miR-210, miR-221 and cytokines (e.g., interleukins: IL-6, IL-8 and IL-10, TNF-α, TGF-β, FGF2 and VEGF) have been found to be proangiogenic factors to promote neovascularization and metastasis [13,105–111]. Exosomal miR-9, for example, is produced by tumor cells and promotes the pathway of Janus kinase/signal transducers and transcription activators by cytokine signaling 5 suppressor levels to promote tumor angiogenesis [112].

Exosomal onco-miR-21 can activate signal transducer and activators of transcription 3 pathway in recipient cells, resulting in increased cellular VEGF receptor levels, showing that miR-21 is essential in angiogenesis. The overexpressed onco-miR-21 exerts its promoting effect on the antiapoptotic protein Bcl-2 and tumor-suppressor protein p53, and its increased concentration is positively connected with metastasis and poor prognosis of breast and lung cancer patients [113].

## Activating invasion & metastasis

Metastasis is a systemic disease that results from a completely malignant tumor’s interaction with both local and distant microenvironments, resulting in secondary malignant development [60,114]. It is well known that the most prevalent cause of cancer-related mortality is metastasis, which accounts for more than 90% of all cancer-related deaths [49,115]. It is a complex process in which cancer cells spread throughout the body from a primary malignant tumor to other organs, producing secondary tumors at the new tissue sites. Several studies have indicated that exosomes derived from tumor cell play a critical role in tumor metastatic pathophysiology by utilizing tumor-signaling pathways such as caveolin-1, HIF-1a, miR-21, miR-105, miR-148b, miR-210, β-catenin and oncogenic kinases (e.g., mutated EGFR, RAS and MAP kinases) [49,116–120].

Tumor-derived exosomes have been found to enhance metastasis at distant organs in addition to modifying the local TME to induce cancer proliferation [119–121]. Tumor metastasis is a multistep process that includes the separation from main organs, invasion, movement via the basement membrane, bloodstream diffusion and eventually adaption and colonization in secondary organ sites [122,123]. Exosome-mediated strategies have been developed by cancer cells to influence a variety of phases in the metastatic process. For example, triple-negative breast cancer cell lines (MDA-MB-231) overexpress miR-10b, and exosomes produced from these cells can transfer miR-10b to a nonmalignant human mammary epithelial cell line, inducing invasion [124]. Exosomes derived from epithelial ovarian cancer cells have been found to promote ovarian cancer invasion by transferring CD44 to human peritoneal mesothelial cells [124]. miR-105 from metastatic MDA-MB-231 exosomes can also target the tight junction protein ZO-1, destroying endothelial cell barriers, causing vascular leakage and promoting metastasis *in vivo* models [125]. Recently, it has been found that the overexpression of tumor-derived exosomal miR-3157-3p promotes angiogenesis, increases vascular permeability and mediates metastasis by downregulating Kruppel-like factor 2 and tissue inhibitor of metalloproteinase 2 in non-small-cell lung cancer in mouse [126].

Cancer cells may influence the microenvironment of the distance organ after invasion and intravasation to allow tumor cells to survive and colonize before they arrive [127]. Exosomes from cancer cells have the ability to initiate such a process [127–130]. Exosomes derived from pancreatic ductal adenocarcinoma have been shown to induce premetastatic niche formation in the liver, and naive mice treated with pancreatic ductal adenocarcinoma exosomes had an increased liver metastatic burden [128]. Exosomes from melanoma have also been shown to modify distant lymph nodes, facilitating the formation of the premetastatic niche [130]. Exosomes from melanoma cells found in sentinel lymph nodes influence the lymph node distribution pattern of free melanoma cells and promote cell migration to exosomes-rich sites.

## Diagnostic & prognostic biomarkers

Exosome biology in disease is still a work in progress, but the number of researches looking at their use in the diagnosis and treatment of a variety of diseases has increased dramatically. The complex contents of exosomes are exploited here, enabling for a multicomponent diagnostic window into disease detection and prognosis monitoring [10]. Exosomes have been shown in several studies to be potential candidates for use as both diagnostic and prognostic biomarkers for various diseases including cancer. Exosomes contain various miRNAs or clusters of miRNAs that can be used to diagnose or prognose cancer [10]. Due to their differential expression between cancer cells and normal cells, oncogenic and tumor-suppressor miRNAs in exosomes may have a high diagnostic value, potentially improving their usefulness in early diagnosis [131].

Glioblastomas, colorectal, colon, liver, breast, ovarian and esophageal cancers have all been linked to elevated circulating exosomal miR-21, whereas bladder and prostate cancers have been linked to elevated urine-derived exosomal miR-21 [10,132]. miR-155, the miR-17–92 cluster and miR-1246 are other exosomal oncogenic miRNAs associated to a variety of cancers [58,133–135]. Upregulation of these miRNAs has been seen in cancers of the brain, pancreas, colorectum, colon, liver, breast, prostate and esophagus, as well as lymphoma and leukemia [10]. Tumor-suppressor miRNAs, including miR-634, miR-146a and miR-34a, have been associated to cancers of the liver, breast, colon, pancreas and hematology [131]. It has been recently reported that the intravenous administration of miR-634 induces cell death and significantly reduces the xenograft tumors growth of pancreatic cancer in mice [136]. This result suggests that delivery of miR-634 can potentially be used for cancer therapy.

Exosomal miRNA biomarkers are constantly emerging in connection with cancer diagnosis and prognosis, and the combination of multiple miRNAs may enhance the diagnostic and prognostic value of exosomal miRNA (Table 1) [62,132,137–144].

**Table 1.** Clinical use of tumor-derived exosome as diagnostic or prognostic biomarkers.

Clinical use	Used sample	Exosomes				Ref.
Diagnostic	Plasma Serum Urine	miR-19-3p miR-629 miR-151a-5p miR-21-5p miR-181a-5p CD151 miR-23a miR-196a-3p	miR-23a-3p miR-205-5p miR-17-3p miR-486-5p let-7d-3p CD171 miR-155 miR-501-3p	miR-361-5p miR-200b miR-361-5p miR-320b miR-30d-5p LRG1 miR-126	miR-200b-5p miR-100 miR-15b-5p miR-30a-3p miR-210-3p tetraspanin8 miR-205	[33,58,61,62,142,145–152]
Prognostic	Plasma Serum	miR-4257 miR-23b-3p miR-23a EGFR miR-21	miR-10b-5p miR-21-5p miR-32 GRB2 miR-146a-5p	miR-208a miR-96 miR-425-3p HSP70 miR-155	miR-222-3p miR-221-3p miR-18a NY-ESO-1	[142,144,153–157]
Therapy	Plasma Serum	miR-221-3p miR-146a-5p	miR-29a miR-150	miR-634		[136,143]

EGFR: Epidermal growth factor receptor; GRB2: Growth factor receptor-bound protein 2; HSP70: Heat shock protein 70; LRG1: Leucine rich alpha-2-glycoprotein 1; NY-ESO-1: Cancer-testis antigen NY-ESO-1.

## Delivery vehicle (drugs, vaccines, proteins & nucleic acids)

Exosomes derived from various sources such as MSCs, tumor cells and immune cells have been used as a vehicle to deliver a large number of molecules, including drugs, nucleic acids,and natural phytochemicals . Biologically active compounds can be found inside exosomes or associated to their surface molecules. Because of their amphiphilic characteristics, they can carry both hydrophobic and hydrophilic molecules.

Various loading methods have been utilized to load therapeutic molecules in this sort of delivery system, including simple incubation, sonication, electroporation, freeze–thaw cycles and extrusion among others, with cells from various types of tissues, such as brain tissue, as target [12,158]. To restore the membranes, one of the physical procedures involves several cycles of sonication followed by incubation at 37°C [151,158,159]. When exosomes are electroporated, the drug molecules penetrate through temporary membrane pores created by electrical pulses [160]. To summarize, hydrophobic molecules penetrate the exosome membrane more easily, whereas hydrophilic compounds have low diffusion efficiency and require modifying the molecule or increasing membrane permeability through physical or chemical methods. When comparing the various methods for encapsulating pharmaceuticals and genetic cargo in exosomes, sonication provides the most efficient encapsulation. The consequences of this treatments on the membrane are a subject that requires more research. In addition, as with isolation techniques, encapsulation procedure standardization, reproducibility and scalability are required for clinical applications. Exosomes containing therapeutic molecules such as drugs and vaccines can be given intravenously, intraperitoneally, intranasally, subcutaneously or orally *in vivo*.

Exosomes have a number of advantages as drug delivery vehicles, including minimal immunogenicity, long-term safety and lack of cytotoxicity [161–163]. Traditional methods of delivering miRNAs, proteins and chemical drugs frequently fail to achieve the desired effects for a variety of reasons: free miRNAs are rapidly destroyed *in vivo*, proteins lack native conformation and chemical drugs are likely to cause high toxicity in normal cells. However, employing exosomes as carriers can protect miRNAs from degradation by ribonuclease within body fluids, therefore exosome-based cargo delivery to tumor cells holds a lot of promise [12,161–163]. Exosomes are being actively explored as therapeutic agents, either on their own or as delivery vehicles for the delivery of drug payloads or vaccines. When administered exogenously in mice, exosomes are more efficient at entering other cells than liposomes and can deliver a functional cargo with less immune clearance [51,164–167]. Exosome enrichment based on their surface ligand presentation could lead to the development of receptor-mediated tissue targeting. Engineered exosomes with ligand enrichment can also be employed to stimulate or inhibit signaling processes in recipient cells, or to target exosomes to certain cell types [12,32,51,164–167].

Exosomes can serve as not only potential biomarkers in medicine but also as a very valuable and effective ‘nanovector’ for delivering anticancer drug compounds to target tissues with minimum immunogenicity and toxicity because of their biological origin when compared with the conventional drug delivery vehicles in cancer therapy [168–170]. Exosomes can be employed as a therapeutic drug delivery vehicle to target cells since they are small, nontoxic, nonimmunogenic and native to humans. Their membrane composition is similar to that of the body’s cell membranes, and have a long life period in the blood circulation system as they protected from degradation by ribonucleases [168]. The characteristic properties of exosomes in delivering functional cargo content to diseased cells make them attractive as therapeutic vehicles at both the fundamental and applied levels. Researchers recently developed natural nanoexosomes, a drug delivery system based on conjugating gold nanoparticles with the anticancer drug doxorubicin and attaching them to the exosome pH-sensitive hydrazone [62]. In another approach, the nanoparticles (e.g. gold, iron oxide and drugs) coated with exosomes have been employed for targeted delivery of therapeutic and imaging agents to specific regions [171,172].

Exosomes that have been engineered to carry drugs or express specific molecules on their surface have opened up new possibilities in the treatment of many diseases. Exosome genetic engineering is a convenient way to give exosomes new characteristics. Exosome surface modification, such as cell transgenic expression, chemical modification, electrostatic interaction and membrane-anchoring platform can increase the targeting and antidisease efficacy [12]. To begin, ligands or homing peptides are fused to exosome surface-expressed transmembrane proteins. LAMP-2B is now the most extensively used exosomal surface protein with a targeting motif. The LAMP-2B belongs to the lysosome-associated membrane protein (LAMP) family that is mostly found in lysosomes and endosomes, with a little amount migrating to the cell surface [173]. Genetic engineering procedures are used to genetically fuse a targeting ligand with an exosome surface membrane protein, which is then overexpressed in target donor cells. As a result, the donor cells produce exosomes that have been genetically modified to carry the targeting ligand. For the display of functional ligands on the exosomal membrane, genetic engineering of exosomes is a highly effective strategy.

Exosomes from different cell origins have different surface molecules anchored on them, allowing them to target certain recipient cells. Surface engineering aims to raise the local concentration of exosomes at the diseased site, lowering toxicity and side effects while increasing therapeutic efficacy. Despite the fact that surface engineering is commonly utilized in targeted drug delivery, little is known about how it affects exosome stability, cellular entry paths and tissue distribution *in vivo* system. Exosomes with large amounts of disease antigens or specific chemokines can successfully recruit antidisease immune cells to disease locations and induce abnormal cell-specific cytotoxicity.

## Conclusion

Exosomes are being investigated as multicomponent signaling complexes that allow communication between nearby and distant tissue cells through one cell to another. They are also being studied as a delivery vehicle for their cargo, which includes proteins, nucleic acids, drugs and vaccines. Exosomes have been shown to be an effective vehicle for cargo delivery due to their natural characteristics of nontoxicity and low immunogenicity, as well as their ability to reach specific target cells at any site within the body due to their small size [15,174–176].

Current findings of the content of healthy and disease-derived exosomes have revealed that these vesicles include a variety of signaling material capable of initiating pathogenesis, as well as their nucleic acid and protein profiles that differ from healthy cell-derived exosomes [60]. Chemical pharmaceuticals, such as RNA therapeutic exosomes, have gradually turned to disease therapy in the clinic. Exosomes with nanotechnological modifications, in particular, have higher biocompatibility and are expected to become a ‘future star’ of therapeutic vaccinations [177].

As a result, exosomes are an excellent delivery system for a variety of diseases, including neurodegeneration, cancer and other diseases, due to their biocompatibility, low toxicity and immunogenicity, high stability in physiological fluids, ability to pass various through biological barriers and ability to load specific molecules (e.g. proteins, miRNAs, antisense oligonucleotides, drugs and vaccines) to targeted cells. Exosomes clearly have innovative and crucial applications in the treatment of diseases, but there are still challenges to overcome. Exosome analysis methods should use small-sample volumes, be rapid, sensitive, specific, high yield, high purity, low cost and clinical validation in order to be helpful in the clinical settings. Purification, concentration, source clarity and sample validation are all critical considerations for employing exosomal miRNA as clinical biomarkers in liquid biopsy. Although the application of exosomes and their biomarkers is currently limited, it is anticipated that clinical use of these biomolecules and their biomarkers will be available shortly.

## Future perspective

In summary, while fascinating exosome biology is being unraveled mostly through cell-culture systems, investigations using *in vivo* models and physiologically relevant experimental circumstances are required. The need for precise and accurate characterization of exosomes will continue to expand as our understanding of exosome heterogeneity, cargo content and roles expands. Animal models to research exosome biogenesis, trafficking and cellular entrance are desperately needed in this field [10].

Despite recent advances in exo-miRNA detection methods, more work is needed to develop more sensitive, rapid, sufficient yield and cost-effective methods for more accurate characterization and functions of body fluid exosomal miRNAs, providing a new strategy for better disease prevention, early and accurate diagnosis, and personalized treatment. The clinical application of engineered exosome delivery for *in vivo* diagnosis, prognosis monitoring and therapy will be greatly facilitated as a result of this.

In summary, exosomes in body fluids have made tremendous progress in the clinic for diagnosing diseases and assessing patient prognosis. However, reliable exosomal biomarkers for early-stage disease detection and prognosis monitoring in large-scale samples that can be adapted to clinical applications, are urgently needed [62]. Although exosome analyses have advanced significantly in recent decades, advancements in exosome detection, separation, purification and profiling procedures are required in order to understand the cargo components, characteristics and functions, which would in turn provide insight on biogenesis. Furthermore, with increased knowledge of exosome biogenesis and function, we will be able to modify exosome composition, characteristics and cell interactions to improve their therapeutic potential. The engineered exosomes will contribute to the creation of novel therapeutic strategies for human therapy as research on exosomes combined with immunotherapy progresses and optimization. Overall, the potential of exosomes derived from numerous sources in terms of disease treatment is highly promising.

**Executive summary**

- Exosomes in body fluids have made tremendous progress in the clinic for diagnosing diseases and assessing patient prognosis.
- Reliable exosomal biomarkers for early-stage disease detection and prognosis monitoring in large-scale samples, that can be adapted to clinical applications, are urgently needed [62].
- Exosome analyses have advanced significantly in recent decades, advancements in exosome detection, separation, purification and profiling procedures are required in order to understand the cargo components, characteristics, and functions, which would in turn provide insight on biogenesis.
- With increased knowledge of exosome biogenesis and function, we will be able to modify exosome composition, characteristics, and cell interactions to improve their therapeutic potential.
- Engineered exosomes will contribute to the creation of novel therapeutic strategies for human therapy as research on exosomes combined with immunotherapy progresses and optimization.
- The potential of exosomes derived from numerous sources in terms of disease treatment is highly promising.

## References

[1] Pan BT, Johnstone RM. Fate of the transferrin receptor during maturation of sheep reticulocytes *in vitro* selective externalization of the receptor. Cell 33, 967–978 (1983). 6307529 10.1016/0092-8674(83)90040-5

[2] Dilsiz N. Role of exosomes and exosomal microRNAs in cancer. Future Sci. OA. 6, 1–15 (2020). 10.2144/fsoa-2019-0116 PMC7117563 32257377

[3] Ludwig AK, Giebel B. Exosomes: small vesicles participating in intercellular communication. Int. J. Biochem. Cell Biol. 44(1), 11–15 (2012). 22024155 10.1016/j.biocel.2011.10.005

[4] Raposo G, Stoorvogel W. Extracellular vesicles exosomes micro vesicles and friends. J. Cell Biol. 200, 373–383 (2013). 23420871 10.1083/jcb.201211138 PMC3575529

[5] Tomasetti M, Lee W, Santarelli L, Neuzil J. Exosome-derived microRNAs in cancer metabolism: possible implications in cancer diagnostics and therapy. Exp. Mol. Med. 49(1), e285–e285 (2017). 28104913 10.1038/emm.2016.153 PMC5291842

[6] Kowal J, Tkach M, Théry C. Biogenesis and secretion of exosomes. Curr. Opin. Cell Biol. 29, 116–125 (2014). 24959705 10.1016/j.ceb.2014.05.004

[7] Dreyer F, Baur A. Biogenesis and functions of exosomes and extracellular vesicles. Methods Mol. Biol. 1448, 201–216 (2016). 27317183 10.1007/978-1-4939-3753-0_15

[8] Wang Y, Xu YM, Zou YQ Identification of differential expressed PE exosomal miRNA in lung adenocarcinoma, tuberculosis, and other benign lesions. Medicine (Baltimore) 96(44), e8361 (2017). 29095265 10.1097/MD.0000000000008361 PMC5682784

[9] Wang B, Xing D, Zhu Y, Dong S, Zhao B. The state of exosomes research: a global visualized analysis. BioMed Res. Int. 2019, 1–10 (2019). 10.1155/2019/1495130 PMC6470441 31073519

[10] Kalluri R, LeBleu VS. The biology, function, and biomedical applications of exosomes. Science 367(6478), eaau6977 (2020). 32029601 10.1126/science.aau6977 PMC7717626

[11] Bhattacharyya K, Mukherjee S. Fluorescent metal nano-clusters as next generation fluorescent probes for cell imaging and drug delivery. Bull. Chem. Soc. Jpn 91(3), 447–454 (2018).

[12] Pi YN, Xia BR, Jin MZ, Jin WL, Lou G. Exosomes: powerful weapon for cancer nano-immunoengineering. Biochem. Pharmacol. 186, 114487 (.2021). 33647264 10.1016/j.bcp.2021.114487

[13] Lu M, Xing H, Xun Z Exosome-based small RNA delivery: progress and prospects. Asian J. Pharm. Sci. 13(1), 1–11 (2018). 32104373 10.1016/j.ajps.2017.07.008 PMC7032220

[14] Wahlgren J, Karlson TDL, Brisslert M Plasma exosomes can deliver exogenous short interfering RNA to monocytes and lymphocytes. Nucleic Acids Res. 40(17), e130–e130 (2012). 22618874 10.1093/nar/gks463 PMC3458529

[15] Samanta S, Rajasingh S, Drosos N Exosomes new molecular targets of diseases. Acta Pharm. Sin. 39, 501–513 (2017). 10.1038/aps.2017.162 PMC5888687 29219950

[16] Cappello F, Logozzi M, Campanella C Exosome levels in human body fluids: a tumor marker by themselves? Eur. J. Pharm. Sci. 96, 93–98 (2017). 27640113 10.1016/j.ejps.2016.09.010

[17] Théry C, Witwer KW, Aikawa E Minimal information for studies of extracellular vesicles 2018 (MISEV2018): a position statement of the International Society for Extracellular Vesicles and update of the MISEV2014 guidelines. J. Extracell. Vesicles 7(1), 1535750 (2018). 30637094 10.1080/20013078.2018.1535750 PMC6322352

[18] Shurtleff MJ, Temoche-Diaz MM, Schekman R. Extracellular vesicles and cancer: caveat lector. Annu. Rev. Cancer Biol. 2, 395–411 (2018).

[19] Jeppesen DK, Fenix AM, Franklin JL Reassessment of exosome composition. Cell 177(2), 428–445 (2019). 30951670 10.1016/j.cell.2019.02.029 PMC6664447

[20] Willms E, Cabañas C, Mäger I, Wood MJ, Vader P. Extracellular vesicle heterogeneity: subpopulations, isolation techniques, and diverse functions in cancer progression. Front. Immunol. 9, 738 (2018). 29760691 10.3389/fimmu.2018.00738 PMC5936763

[21] Gurung S, Perocheau D, Touramanidou L, Baruteau J. The exosome journey: from biogenesis to uptake and intracellular signalling. Cell Commun. Signal. 19(1), 1–19 (2021). 33892745 10.1186/s12964-021-00730-1 PMC8063428

[22] Buschow SI, Anderton SM, Stoorvogel W, Wauben MH. Activated T cells recruit exosomes secreted by dendritic cells via LFA-1. Blood 113(9), 1977–1981 (2009). 19064723 10.1182/blood-2008-08-174094

[23] Ratajczak J, Miekus K, Kucia M Embryonic stem cell-derived microvesicles reprogram hematopoietic progenitors: evidence for horizontal transfer of mRNA and protein delivery. Leukemia 20(5), 847–856 (2006). 16453000 10.1038/sj.leu.2404132

[24] Zhang B, Wang M, Gong A HucMSC-exosome mediated-Wnt4 signaling is required for cutaneous wound healing. Stem Cells 33(7), 2158–2168 (2015). 24964196 10.1002/stem.1771

[25] Cui X, He Z, Liang Z, Chen Z, Wang H, Zhang J. Exosomes from adipose-derived mesenchymal stem cells protect the myocardium against ischemia/reperfusion injury through Wnt/β-catenin signaling pathway. J. Cardiovasc. Pharmacol. 70(4), 225 (2017). 28582278 10.1097/FJC.0000000000000507 PMC5642342

[26] Men Y, Yelick J, Jin S Exosome reporter mice reveal the involvement of exosomes in mediating neuron to astroglia communication in the CNS. Nat. Commun. 10(1), 1–18 (2019). 31515491 10.1038/s41467-019-11534-w PMC6742670

[27] Bang C, Batkai S, Dangwal S Cardiac fibroblast-derived microRNA passenger strand-enriched exosomes mediate cardiomyocyte hypertrophy. J. Clin. Invest. 124(5), 2136–2146 (2014). 24743145 10.1172/JCI70577 PMC4001534

[28] Zamani P, Fereydouni N, Butler AE, Navashenaq JG, Sahebkar A. The therapeutic and diagnostic role of exosomes in cardiovascular diseases. Trends Cardiovasc. Med. 29(6), 313–323 (2019). 30385010 10.1016/j.tcm.2018.10.010

[29] Howitt J, Hill AF. Exosomes in the pathology of neurodegenerative diseases. J. Biol. Chem. 291(52), 26589–26597 (2016). 27852825 10.1074/jbc.R116.757955 PMC5207170

[30] Osaki M, Okada F. Exosomes and their role in cancer progression. Yonago Acta. Medica. 62(2), 182–190 (2019). 31320822 10.33160/yam.2019.06.002 PMC6584259

[31] Deng ZB, Liu Y, Liu C Immature myeloid cells induced by a high-fat diet contribute to liver inflammation. Hepatology 50(5), 1412–1420 (2009). 19708080 10.1002/hep.23148 PMC2852608

[32] Hade MD, Suire CS, Suo Z. Mesenchymal stem cell-derived exosomes: applications in regenerative medicine. Cells 10, 1959 (2021). 34440728 10.3390/cells10081959 PMC8393426

[33] Zhang Y, Liu Y, Liu H, Tang WH. Exosomes: biogenesis, biologic function and clinical potential. Cell Biosci. 9(1), 1–18 (2019). 30815248 10.1186/s13578-019-0282-2 PMC6377728

[34] Haney MJ, Klyachko NL, Zhao Y Exosomes as drug delivery vehicles for Parkinson's disease therapy. J. Control. Rel. 207, 18–30 (2015). 10.1016/j.jconrel.2015.03.033 PMC4430381 25836593

[35] Bunggulawa EJ, Wang W, Yin T Recent advancements in the use of exosomes as drug delivery systems. Nanobiotechnology 16(1), 1–13 (2018). 10.1186/s12951-018-0403-9 PMC6190562 30326899

[36] Wu J, Shen Z. Exosomal miRNAs as biomarkers for diagnostic and prognostic in lung cancer. Cancer Med. 9(19), 6909–6922 (2020). 32779402 10.1002/cam4.3379 PMC7541138

[37] Thakur A, Parra DC, Motallebnejad P, Brocchi M, Chen HJ. Exosomes: small vesicles with big roles in cancer, vaccine development, and therapeutics. Bioact. Mater. 9, 1–14 (2021). 34901546 10.1016/j.bioactmat.2021.08.029 PMC8636666

[38] Adlakha YK, Seth P. The expanding horizon of microRNAs in cellular reprogramming. Prog. Neurobiol. 148, 21–39 (2017). 27979736 10.1016/j.pneurobio.2016.11.003

[39] Yao Q, Chen Y, Zhou X. The roles of microRNAs in epigenetic regulation. Curr. Opin. Chem. Biol. 51, 11–17 (2019). 30825741 10.1016/j.cbpa.2019.01.024

[40] Moutinho C, Esteller M. MicroRNAs and epigenetics. Adv. Cancer Res. 135, 189–220 (2017). 28882223 10.1016/bs.acr.2017.06.003

[41] Fabbri M, Garzon R, Cimmino A MicroRNA-29 family reverts aberrant methylation in lung cancer by targeting DNA methyltransferases 3A and 3B. Proc. Natl Acad. Sci. 104(40), 15805–15810 (2007). 17890317 10.1073/pnas.0707628104 PMC2000384

[42] Garzon R, Liu S, Fabbri M MicroRNA-29b induces global DNA hypomethylation and tumor suppressor gene reexpression in acute myeloid leukemia by targeting directly DNMT3A and 3B and indirectly DNMT1. Blood 113(25), 6411–6418 (2009). 19211935 10.1182/blood-2008-07-170589 PMC2710934

[43] Tu J, Liao J, Luk AC, Tang NL, Chan WY, Lee TL. MicroRNAs mediated targeting on the Yin-yang dynamics of DNA methylation in disease and development. Int. J. Biochem. Cell Biol. 67, 115–120 (2015). 25979370 10.1016/j.biocel.2015.05.002

[44] Taguchi YH. Apparent microRNA-target-specific histone modification in mammalian spermatogenesis. Evol. Bioinform. Online 11(Suppl. 1), 13–26 (2015). 25780334 10.4137/EBO.S21832 PMC4345942

[45] Mens MM, Ghanbari M. Cell cycle regulation of stem cells by microRNAs. Stem Cell Rev. Rep. 14(3), 309–322 (2018). 29541978 10.1007/s12015-018-9808-y PMC5960494

[46] Hao J, Duan FF, Wang Y. MicroRNAs and RNA binding protein regulators of microRNAs in the control of pluripotency and reprogramming. Curr. Opin. Genet. Dev. 46, 95–103 (2017). 28753462 10.1016/j.gde.2017.07.001

[47] Li XJ, Ren ZJ, Tang JH, Yu Q. Exosomal MicroRNA MiR-1246 promotes cell proliferation, invasion and drug resistance by targeting CCNG2 in breast cancer. Cell. Physiol. Biochem. 44(5), 1741–1748 (2017). 29216623 10.1159/000485780

[48] Lichner Z, Páll E, Kerekes A The miR-290-295 cluster promotes pluripotency maintenance by regulating cell cycle phase distribution in mouse embryonic stem cells. Differentiation 81(1), 11–24 (2011). 20864249 10.1016/j.diff.2010.08.002

[49] Markopoulos GS, Roupakia E, Tokamani M A step-by-step microRNA guide to cancer development and metastasis. Cellular Onco. 40(4), 303–339 (2017). 10.1007/s13402-017-0341-9 PMC13001585 28748501

[50] Antonyak MA, Cerione RA. Micro vesicles as mediators of intercellular communication in cancer. Methods Mol. Biol. 1165, 147–173 (2014). 24839024 10.1007/978-1-4939-0856-1_11

[51] Kalluri R. The biology and function of exosomes in cancer. J. Clin. Investig. 126(4), 1208–1215 (2016). 27035812 10.1172/JCI81135 PMC4811149

[52] Mathieu M, Martin-Jaular L, Lavieu G, Théry C. Specificities of secretion and uptake of exosomes and other extracellular vesicles for cell-to-cell communication. Nat. Cell Biol. 21(1), 9–17 (2019). 30602770 10.1038/s41556-018-0250-9

[53] Baretti M, Le DT. DNA mismatch repair in cancer. Pharmacol. Ther. 189, 45–62 (2018). 29669262 10.1016/j.pharmthera.2018.04.004

[54] Judson RL, Babiarz JE, Venere M, Blelloch R. Embryonic stem cell–specific microRNAs promote induced pluripotency. Nat. Biotechnol. 27(5), 459–461 (2009). 19363475 10.1038/nbt.1535 PMC2743930

[55] Lin SL, Chang DC, Chang-Lin S Mir-302 reprograms human skin cancer cells into a pluripotent ES-cell-like state. RNA 14(10), 2115–2124 (2008). 18755840 10.1261/rna.1162708 PMC2553732

[56] Miyoshi N, Ishii H, Nagano H Reprogramming of mouse and human cells to pluripotency using mature microRNAs. Cell Stem Cell 8(6), 633–638 (2011). 21620789 10.1016/j.stem.2011.05.001

[57] Samavarchi-Tehrani P, Golipour A, David L. Functional genomics reveals a BMP-driven mesenchymal-to-epithelial transition in the initiation of somatic cell reprogramming. Cell Stem Cell 7(1), 64–77 (2010). 20621051 10.1016/j.stem.2010.04.015

[58] Li N, Long B, Han W, Yuan S, Wang K. microRNAs: important regulators of stem cells. Stem Cell Res. Ther. 8(1), 1–7 (2017). 28494789 10.1186/s13287-017-0551-0 PMC5426004

[59] Zeng ZL, Lin XL, Tan LL, Liu YM, Qu K, Wang Z. MicroRNAs: important regulators of induced pluripotent stem cell generation and differentiation. Stem Cell Rev. Rep. 14(1), 71–81 (2018). 29143183 10.1007/s12015-017-9785-6

[60] Stefanius K, Servage K, Orth K. Exosomes in cancer development. Curr. Opin. Genet. Dev. 66, 83–92 (2021). 33477017 10.1016/j.gde.2020.12.018

[61] Xue X, Wang X, Zhao Y, Hu R, Qin L. Exosomal miR-93 promotes proliferation and invasion in hepatocellular carcinoma by directly inhibiting TIMP2/TP53INP1/CDKN1A. Biochem. Biophys. Res. Commun. 502(4), 515–521 (2018). 29859935 10.1016/j.bbrc.2018.05.208

[62] Xu K, Zhang C, Du T Progress of exosomes in the diagnosis and treatment of lung cancer. Biomed. Pharmacother. 134, 111111 (2021). 33352449 10.1016/j.biopha.2020.111111

[63] Sun Z, Shi K, Yang S Effect of exosomal miRNA on cancer biology and clinical applications. Mol. Cancer 17(1), 1–19 (2018). 30309355 10.1186/s12943-018-0897-7 PMC6182840

[64] Li X, Corbett AL, Taatizadeh E Challenges and opportunities in exosome research-perspectives from biology, engineering, and cancer therapy. APL Bioeng. 3(1), 011503 (2019). 31069333 10.1063/1.5087122 PMC6481742

[65] Meehan K, Vella LJ. The contribution of tumour-derived exosomes to the hallmarks of cancer. Crit. Rev. Clin. Lab. Sci. 53(2), 121–131 (2016). 26479834 10.3109/10408363.2015.1092496

[66] Qu JL, Qu XJ, Zhao MF Gastric cancer exosomes promote tumour cell proliferation through PI3K/Akt and MAPK/ERK activation. Dig. Liver Dis. 41(12), 875–880 (2009). 19473897 10.1016/j.dld.2009.04.006

[67] Li C, Liu DR, Li GG CD97 promotes gastric cancer cell proliferation and invasion through exosome-mediated MAPK signaling pathway. World J. Gastroenterol. 21(20), 6215 (2015). 26034356 10.3748/wjg.v21.i20.6215 PMC4445098

[68] Yang L, Wu XH, Wang D, Luo CL, Chen LX. Bladder cancer cell-derived exosomes inhibit tumor cell apoptosis and induce cell proliferation *in vitro*. Mol. Med. Rep. 8(4), 1272–1278 (2013). 23969721 10.3892/mmr.2013.1634

[69] Sento S, Sasabe E, Yamamoto T. Application of a persistent heparin treatment inhibits the malignant potential of oral squamous carcinoma cells induced by tumor cell-derived exosomes. PLoS ONE 11(2), e0148454 (2016). 26849680 10.1371/journal.pone.0148454 PMC4743844

[70] Janowska-Wieczorek A, Wysoczynski M, Kijowski J Microvesicles derived from activated platelets induce metastasis and angiogenesis in lung cancer. Int. J. Cancer 113(5), 752–760 (2005). 15499615 10.1002/ijc.20657

[71] Webber J, Steadman R, Mason MD, Tabi Z, Clayton A. Cancer exosomes trigger fibroblast to myofibroblast differentiation. Cancer Res. 70(23), 9621–9630 (2010). 21098712 10.1158/0008-5472.CAN-10-1722

[72] Webber JP, Spary LK, Sanders AJ Differentiation of tumour-promoting stromal myofibroblasts by cancer exosomes. Oncogene 34(3), 290–302 (2015). 24441045 10.1038/onc.2013.560

[73] Kalluri R, Zeisberg M. Fibroblasts in cancer. Nat. Rev. Cancer 6(5), 392–401 (2006). 16572188 10.1038/nrc1877

[74] Goulet CR, Bernard G, Tremblay S, Chabaud S, Bolduc S, Pouliot F. Exosomes induce fibroblast differentiation into cancer-associated fibroblasts through TGFβ signaling. Mol. Cancer Res. 16(7), 1196–1204 (2018). 29636362 10.1158/1541-7786.MCR-17-0784

[75] Melo SA, Sugimoto H, O'Connell JT Cancer exosomes perform cell-independent microRNA biogenesis and promote tumorigenesis. Cancer Cell 26(5), 707–721 (2014). 25446899 10.1016/j.ccell.2014.09.005 PMC4254633

[76] Abd Elmageed ZY, Yang Y, Thomas R Neoplastic reprogramming of patient-derived adipose stem cells by prostate cancer cell-associated exosomes. Stem Cells 32(4), 983–997 (2014). 24715691 10.1002/stem.1619 PMC4184251

[77] Marson A, Levine SS, Cole MF Connecting microRNA genes to the core transcriptional regulatory circuitry of embryonic stem cells. Cell 134(3), 521–533 (2008). 18692474 10.1016/j.cell.2008.07.020 PMC2586071

[78] Sluijter JP, van Mil A, van Vliet P. MicroRNA-1 and-499 regulate differentiation and proliferation in human-derived cardiomyocyte progenitor cells. Arterioscler. Thromb. Vasc. Biol. 30(4), 859–868 (2010). 20081117 10.1161/ATVBAHA.109.197434

[79] Glass C, Singla DK. MicroRNA-1 transfected embryonic stem cells enhance cardiac myocyte differentiation and inhibit apoptosis by modulating the PTEN/Akt pathway in the infarcted heart. Am. J. Physiol. Heart Circ. Physiol. 301(5), H2038–H2049 (2011). 21856911 10.1152/ajpheart.00271.2011 PMC3213958

[80] Xie C, Huang H, Sun X MicroRNA-1 regulates smooth muscle cell differentiation by repressing Kruppel-like factor 4. Stem Cells Dev. 20(2), 205–210 (2011). 20799856 10.1089/scd.2010.0283 PMC3128754

[81] Chen T, Margariti A, Kelaini S MicroRNA-199b modulates vascular cell fate during iPS cell differentiation by targeting the notch ligand jagged1 and enhancing VEGF signaling. Stem Cells 33(5), 1405–1418 (2015). 25535084 10.1002/stem.1930 PMC4737258

[82] Cheng LC, Pastrana E, Tavazoie M, Doetsch F. miR-124 regulates adult neurogenesis in the subventricular zone stem cell niche. Nat. Neurosci. 12(4), 399 (2009). 19287386 10.1038/nn.2294 PMC2766245

[83] Agostini M, Tucci P, Steinert JR microRNA-34a regulates neurite outgrowth, spinal morphology, and function. Proc. Natl Acad. Sci. USA 108(52), 21099–21104 (2011). 22160706 10.1073/pnas.1112063108 PMC3248521

[84] Kim H, Lee G, Ganat Y miR-371-3 expression predicts neural differentiation propensity in human pluripotent stem cells. Cell Stem Cell 8(6), 695–706 (2011). 21624813 10.1016/j.stem.2011.04.002

[85] Eskildsen T, Taipaleenmäki H, Stenvang J MicroRNA-138 regulates osteogenic differentiation of human stromal (mesenchymal) stem cells *in vivo*. Proc. Natl Acad. Sci. USA 108(15), 6139–6144 (2011). 21444814 10.1073/pnas.1016758108 PMC3076836

[86] Ham O, Song BW, Lee SY The role of microRNA-23b in the differentiation of MSC into chondrocyte by targeting protein kinase A signaling. Biomaterials 33(18), 4500–4507 (2012). 22449550 10.1016/j.biomaterials.2012.03.025

[87] Lin X, Wu L, Zhang Z MiR-335-5p promotes chondrogenesis in mouse mesenchymal stem cells and is regulated through two positive feedback loops. J. Bone Miner. Res. 29(7), 1575–1585 (2014). 24347469 10.1002/jbmr.2163

[88] Meenhuis A, van Veelen PA, de Looper H MiR-17/20/93/106 promote hematopoietic cell expansion by targeting sequestosome 1-regulated pathways in mice. Blood 118(4), 916–925 (2011). 21628417 10.1182/blood-2011-02-336487 PMC3148171

[89] Chen CZ, Li L, Lodish HF, Bartel DP. MicroRNAs modulate hematopoietic lineage differentiation. Science 303(5654), 83–86 (2004). 14657504 10.1126/science.1091903

[90] Wang X, Zhou Y, Ding K. Roles of exosomes in cancer chemotherapy resistance, progression, metastasis and immunity, and their clinical applications. Int. J. Oncol. 59(1), 1–18 (2021). 10.3892/ijo.2021.5224 PMC8143748 34013358

[91] Zhao J, Schlößer HA, Wang Z Tumor-derived extracellular vesicles inhibit natural killer cell function in pancreatic cancer. Cancers 11(6), 874 (2019). 10.3390/cancers11060874 PMC6628179 31234517

[92] Chen G, Huang AC, Zhang W Exosomal PD-L1 contributes to immunosuppression and is associated with anti-PD-1 response. Nature 560(7718), 382–386 (2018). 30089911 10.1038/s41586-018-0392-8 PMC6095740

[93] Théry C, Ostrowski M, Segura E. Membrane vesicles as conveyors of immune responses. Nat. Rev. Immunol. 9(8), 581–593 (2009). 19498381 10.1038/nri2567

[94] Barros FM, Carneiro F, Machado JC, Melo SA. Exosomes and immune response in cancer: friends or foes? Front. Immunol. 9, 730 (2018). 29696022 10.3389/fimmu.2018.00730 PMC5904196

[95] Quah BJ, O'Neill HC. The immunogenicity of dendritic cell-derived exosomes. Blood Cells Mol. Dis. 35(2), 94–110 (2005). 15975838 10.1016/j.bcmd.2005.05.002

[96] Romagnoli GG, Zelante BB, Toniolo PA, Migliori IK, Barbuto JAM. Dendritic cell-derived exosomes may be a tool for cancer immunotherapy by converting tumor cells into immunogenic targets. Front. Immunol. 5, 692 (2015). 25646096 10.3389/fimmu.2014.00692 PMC4298225

[97] Kugeratski FG, Kalluri R. Exosomes as mediators of immune regulation and immunotherapy in cancer. FEBS J. 288(1), 10–35 (2021). 32910536 10.1111/febs.15558 PMC9116040

[98] Fernandes Ribeiro M, Zhu H, W Millard R, Fan GC. Exosomes function in pro-and anti-angiogenesis. Curr. Angiogenes. 2(1), 54–59 (2013). 25374792 10.2174/22115528113020020001 PMC4217212

[99] Martial S. Involvement of ion channels and transporters in carcinoma angiogenesis and metastasis. Am. J. Physiol. Cell Physiol. 310, C710–C727 (2016). 26791487 10.1152/ajpcell.00218.2015

[100] Umezu T, Ohyashiki K, Kuroda M, Ohyashiki JH. Leukemia cell to endothelial cell communication via exosomal miRNAs. Oncogene 32(22), 2747–2755 (2013). 22797057 10.1038/onc.2012.295

[101] Sharghi-Namini S, Tan E, Ong LLS, Ge R, Asada HH. Dll4-containing exosomes induce capillary sprout retraction in a 3D microenvironment. Sci. Rep. 4(1), 1–8 (2014). 10.1038/srep04031 PMC3916896 24504253

[102] Dorayappan KDP, Wanner R, Wallbillich JJ Hypoxia-induced exosomes contribute to a more aggressive and chemoresistant ovarian cancer phenotype: a novel mechanism linking STAT3/Rab proteins. Oncogene 37(28), 3806–3821 (2018). 29636548 10.1038/s41388-018-0189-0 PMC6043362

[103] Mao Y, Wang Y, Dong L Hypoxic exosomes facilitate angiogenesis and metastasis in esophageal squamous cell carcinoma through altering the phenotype and transcriptome of endothelial cells. J. Exp. Clin. Cancer Res. 38(1), 1–14 (2019). 31488217 10.1186/s13046-019-1384-8 PMC6727585

[104] Ludwig N, Whiteside TL. Potential roles of tumor-derived exosomes in angiogenesis. Expert Opin. Ther. Targets 22(5), 409–417 (2018). 29634426 10.1080/14728222.2018.1464141 PMC6126896

[105] Zhou W, Fong MY, Min Y Cancer-secreted miR-105 destroys vascular endothelial barriers to promote metastasis. Cancer Cell 25(4), 501–515 (2014). 24735924 10.1016/j.ccr.2014.03.007 PMC4016197

[106] Umezu T, Tadokoro H, Azuma K, Yoshizawa S, Ohyashiki K, Ohyashiki JH. Exosomal miR-135b shed from hypoxic multiple myeloma cells enhances angiogenesis by targeting factor-inhibiting HIF-1. Blood 124(25), 3748–3757 (2014). 25320245 10.1182/blood-2014-05-576116 PMC4263983

[107] Martinelli C, Farooqi AA, Ismail M. Exosomes new biomarkers for targeted cancer therapy. In: Molecular Oncology Underlying Mechanisms and Translational Advancements AA Farooqi, M Ismail, Springer International Publishing, 129–157 (2017).

[108] Mentkowski KI, Snitzer JD, Rusnak S, Lang JK. Therapeutic potential of engineered extracellular vesicles. AAPS J. 20(3), 1–17 (2018). 10.1208/s12248-018-0211-z PMC8299397 29546642

[109] Ma T, Chen Y, Chen Y MicroRNA-132, delivered by mesenchymal stem cell-derived exosomes, promote angiogenesis in myocardial infarction. Stem Cells Int. 2018, 3290372 (2018). 30271437 10.1155/2018/3290372 PMC6151206

[110] Fortunato O, Gasparini P, Boeri M, Sozzi G. Exo-miRNAs as a new tool for liquid biopsy in lung cancer. Cancers 11(6), 888 (2019). 10.3390/cancers11060888 PMC6627875 31242686

[111] Deng T, Zhang H, Yang H Exosome miR-155 derived from gastric carcinoma promotes angiogenesis by targeting the c-MYB/VEGF axis of endothelial cells. Mol. Ther. Nucleic Acids 19, 1449–1459 (2020). 32160713 10.1016/j.omtn.2020.01.024 PMC7056628

[112] Zhuang G, Wu X, Jiang Z Tumour-secreted miR-9 promotes endothelial cell migration and angiogenesis by activating the JAK-STAT pathway. EMBO J. 31(17), 3513–3523 (2012). 22773185 10.1038/emboj.2012.183 PMC3433782

[113] Wu H, Wang Q, Zhong H Differentially expressed microRNAs in exosomes of patients with breast cancer revealed by next-generation sequencing. Oncol. Rep. 43(1), 240–250 (2020). 31746410 10.3892/or.2019.7401 PMC6908931

[114] Alečković M, McAllister SS, Polyak K. Metastasis as a systemic disease: molecular insights and clinical implications. Biochim. Biophys. Acta Rev. Cancer 1872(1), 89–102 (2019). 31202687 10.1016/j.bbcan.2019.06.002 PMC6692219

[115] Clancy J, D'Souza-Schorey C. Extracellular vesicles in cancer purpose and promise. Cancer J. 24, 65–69 (2018). 29601332 10.1097/PPO.0000000000000306 PMC6645395

[116] Melo SA, Luecke LB, Kahlert C Glypican-1 identifies cancer exosomes and detects early pancreatic cancer. Nature 523(7559), 177–182 (2015). 26106858 10.1038/nature14581 PMC4825698

[117] Sharma A, Khatun Z, Shiras A. Tumor exosomes cellular postmen of cancer diagnosis and personalized therapy. J. Nanomedicine 11, 421–437 (2016). 10.2217/nnm.15.210 26784674

[118] Syn N, Wang L, Sethi G, Thiery JP, Goh BC. Exosome-mediated metastasis: from epithelial–mesenchymal transition to escape from immunosurveillance. Trends Pharmacol. Sci. 37(7), 606–617 (2016). 27157716 10.1016/j.tips.2016.04.006

[119] Wang N, Xie L. Diagnostic and therapeutic applications of tumorassociated exosomes. Precision Rad. Oncol. 1, 34–39 (2017).

[120] Zhang J, Li D, Zhang Y, Ding Z Integrative analysis of mRNA and miRNA expression profiles reveals seven potential diagnostic biomarkers for non-small cell lung cancer. Oncol. Rep. 43(1), 99–112 (2020). 31746439 10.3892/or.2019.7407 PMC6908938

[121] O'Neill S, O'Driscoll L. Profiling circulating miRNAs from the plasma of individuals with metabolic syndrome. Methods Mol. Biol. 13, 141–149 (2017). 10.1007/978-1-4939-6524-3_13 27826924

[122] Dong H, Chen H, Jiang J, Zhang H, Cai C, Shen Q. Highly sensitive electrochemical detection of tumor exosomes based on aptamer recognition-induced multi-DNA release and cyclic enzymatic amplification. Anal. Chem. 90(7), 4507–4513 (2018). 29512380 10.1021/acs.analchem.7b04863

[123] Sitar S, Kejžar A, Pahovnik D Size characterization and quantification of exosomes by asymmetrical-flow field-flow fractionation. Anal. Chem. 87(18), 9225–9233 (2015). 26291637 10.1021/acs.analchem.5b01636

[124] Van der Pol E, Coumans FAW, Grootemaat AE Particle size distribution of exosomes and microvesicles determined by transmission electron microscopy, flow cytometry, nanoparticle tracking analysis, and resistive pulse sensing. J. Thromb. Haemost. 12(7), 1182–1192 (2014). 24818656 10.1111/jth.12602

[125] Maas SL, Broekman ML, de Vrij J. Tunable resistive pulse sensing for the characterization of extracellular vesicles. In: Exosomes and Micro Vesicles. Hill AF (Ed.). **1545**: 21–33; Humana Press, NY (2017). 10.1007/978-1-4939-6728-5_2 27943204

[126] Ma Z, Wei K, Yang F Tumor-derived exosomal miR-3157-3p promotes angiogenesis, vascular permeability and metastasis by targeting TIMP/KLF2 in non-small cell lung cancer. Cell Death Dis. 12(9), 1–13 (2021). 34497265 10.1038/s41419-021-04037-4 PMC8426367

[127] Peinado H, Zhang H, Matei IR Pre-metastatic niches: organ-specific homes for metastases. Nat. Rev. Cancer 17(5), 302 (2017). 28303905 10.1038/nrc.2017.6

[128] Costa-Silva B, Aiello NM, Ocean AJ Pancreatic cancer exosomes initiate pre-metastatic niche formation in the liver. Nat. Cell Biol. 17(6), 816–826 (2015). 25985394 10.1038/ncb3169 PMC5769922

[129] Plebanek MP, Angeloni NL, Vinokour E Pre-metastatic cancer exosomes induce immune surveillance by patrolling monocytes at the metastatic niche. Nat. Commun. 8(1), 1–12 (2017). 29105655 10.1038/s41467-017-01433-3 PMC5673063

[130] Hood JL, San RS, Wickline SA. Exosomes released by melanoma cells prepare sentinel lymph nodes for tumor metastasis. Cancer Res. 71(11), 3792–3801 (2011). 21478294 10.1158/0008-5472.CAN-10-4455

[131] Salehi M, Sharifi M. Exosomal miRNAs as novel cancer biomarkers: challenges and opportunities. J. Cell. Physiol. 233(9), 6370–6380 (2018). 29323722 10.1002/jcp.26481 PMC13482446

[132] Thind A, Wilson C. Exosomal miRNAs as cancer biomarkers and therapeutic targets. J. Extracell. Vesicles 5(1), 31292 (2016). 27440105 10.3402/jev.v5.31292 PMC4954869

[133] Bhagirath D, Yang TL, Bucay N microRNA-1246 is an exosomal biomarker for aggressive prostate cancer. Cancer Res. 78(7), 1833–1844 (2018). 29437039 10.1158/0008-5472.CAN-17-2069 PMC5890910

[134] Pigati L, Yaddanapudi SC, Iyengar R Selective release of microRNA species from normal and malignant mammary epithelial cells. PloS ONE 5(10), e13515 (2010). 20976003 10.1371/journal.pone.0013515 PMC2958125

[135] Sakha S, Muramatsu T, Ueda K, Inazawa J. Exosomal microRNA miR-1246 induces cell motility and invasion through the regulation of DENND2D in oral squamous cell carcinoma. Sci. Rep. 6(1), 1–11 (2016). 27929118 10.1038/srep38750 PMC5144099

[136] Gokita K, Inoue J, Ishihara H, Kojima K, Inazawa J. Therapeutic potential of LNP-mediated delivery of miR-634 for cancer therapy. Mol. Ther. Nucleic Acids 19, 330–338 (2020). 31877409 10.1016/j.omtn.2019.10.045 PMC6938807

[137] Alhasan AH, Scott AW, Wu JJ Circulating microRNA signature for the diagnosis of very high-risk prostate cancer. Proceedings of the National Academy of Sciences. 113(38), 10655–10660 (2016). 10.1073/pnas.1611596113 PMC5035901 27601638

[138] Ebrahimkhani S, Vafaee F, Hallal S Deep sequencing of circulating exosomal microRNA allows non-invasive glioblastoma diagnosis. NPJ Precis. Oncol. 2(1), 1–9 (2018). 30564636 10.1038/s41698-018-0071-0 PMC6290767

[139] Halvaei S, Daryani S, Eslami SZ Exosomes in cancer liquid biopsy: a focus on breast cancer. Mol. Ther. Nucleic Acids 10, 131–141 (2018). 29499928 10.1016/j.omtn.2017.11.014 PMC5862028

[140] Stevic I, Müller V, Weber K Specific microRNA signatures in exosomes of triple-negative and HER2-positive breast cancer patients undergoing neoadjuvant therapy within the GeparSixto trial. BMC Med. 16(1), 1–16 (2018). 10.1186/s12916-018-1163-y PMC6178264 30301470

[141] Zhou X, Zhu W, Li H Diagnostic value of a plasma microRNA signature in gastric cancer: a microRNA expression analysis. Sci. Rep. 5(1), 1–13 (2015). 10.1038/srep11251 PMC4462022 26059512

[142] Jin X, Chen Y, Chen H Evaluation of tumor-derived exosomal miRNA as potential diagnostic biomarkers for early-stage non-small-cell lung cancer using next-generation sequencing. Clin. Cancer Res. 23(17), 5311–5319 (2017). 28606918 10.1158/1078-0432.CCR-17-0577

[143] Dinh TKT, Fendler W, Chałubińska-Fendler J Circulating miR-29a and miR-150 correlate with delivered dose during thoracic radiation therapy for non-small cell lung cancer. Radiat. Oncol. 11(1), 1–11 (2016). 27117590 10.1186/s13014-016-0636-4 PMC4847218

[144] Zhou X, Wen W, Shan X A six-microRNA panel in plasma was identified as a potential biomarker for lung adenocarcinoma diagnosis. Oncotarget 8(4), 6513 (2017). 28036284 10.18632/oncotarget.14311 PMC5351649

[145] Rabinowits G, Gerçel-Taylor C, Day JM, Taylor DD, Kloecker GH. Exosomal microRNA: a diagnostic marker for lung cancer. Clin. Lung Cancer 10(1), 42–46 (2009). 19289371 10.3816/CLC.2009.n.006

[146] Grimolizzi F, Monaco F, Leoni F Exosomal miR-126 as a circulating biomarker in non-small-cell lung cancer regulating cancer progression. Sci. Rep. 7(1), 1–12 (2017). 29127370 10.1038/s41598-017-15475-6 PMC5681649

[147] Xu ZH, Miao ZW, Jiang QZ Brain microvascular endothelial cell exosome-mediated S100A16 up-regulation confers small-cell lung cancer cell survival in brain. FASEB J. 33(2), 1742–1757 (2019). 30183374 10.1096/fj.201800428R

[148] Wang J, Yeung BZ, Cui M Exosome is a mechanism of intercellular drug transfer: application of quantitative pharmacology. J. Control. Rel. 268, 147–158 (2017). 10.1016/j.jconrel.2017.10.020 PMC5722714 29054369

[149] Lai X, Friedman A. Exosomal miRs in lung cancer: a mathematical model. PLoS ONE 11(12), e0167706 (2016). 28002496 10.1371/journal.pone.0167706 PMC5176278

[150] Manier S, Liu CJ, Avet-Loiseau H Prognostic role of circulating exosomal miRNAs in multiple myeloma. Blood 129(17), 2429–2436 (2017). 28213378 10.1182/blood-2016-09-742296 PMC5409448

[151] Kim MS, Haney MJ, Zhao Y Engineering macrophage-derived exosomes for targeted paclitaxel delivery to pulmonary metastases: *in vitro* and *in vivo* evaluations. Nanomedicine 14(1), 195–204 (2018). 28982587 10.1016/j.nano.2017.09.011

[152] Bernard S, Agustriawan D. Identification of microRNA targeting cancer gene of colorectal carcinoma in caucasian population. 2019 International Conference on Information and Communications Technology (ICOIACT). IEEE, 423–427 (2019).

[153] Hsu YL, Hung JY, Chang WA Hypoxic lung cancer-secreted exosomal miR-23a increased angiogenesis and vascular permeability by targeting prolyl hydroxylase and tight junction protein ZO-1. Oncogene 36(34), 4929–4942 (2017). 28436951 10.1038/onc.2017.105

[154] Yuwen D, Ma Y, Wang D Prognostic role of circulating exosomal miR-425-3p for the response of NSCLC to platinum-based chemotherapy. Cancer Epidemiol. Biomarkers Prev. 28(1), 163–173 (2019). 30228154 10.1158/1055-9965.EPI-18-0569

[155] Dejima H, Iinuma H, Kanaoka R, Matsutani N, Kawamura M. Exosomal microRNA in plasma as a non-invasive biomarker for the recurrence of non-small cell lung cancer. Oncol. Lett. 13(3), 1256–1263 (2017). 28454243 10.3892/ol.2017.5569 PMC5403401

[156] Clark DJ, Fondrie WE, Yang A, Mao L. Triple SILAC quantitative proteomic analysis reveals differential abundance of cell signaling proteins between normal and lung cancer-derived exosomes. J. Proteomics 133, 161–169 (2016). 26739763 10.1016/j.jprot.2015.12.023

[157] Kumata Y, Iinuma H, Suzuki Y Exosome-encapsulated microRNA-23b as a minimally invasive liquid biomarker for the prediction of recurrence and prognosis of gastric cancer patients in each tumor stage. Oncol. Rep. 40(1), 319–330 (2018). 29749537 10.3892/or.2018.6418

[158] Gutierrez-Millan C, Calvo Díaz C, Lanao JM, Colino CI. Advances in exosomes-based drug delivery systems. Macromol. Biosci. 21(1), 2000269 (2021). 10.1002/mabi.202000269 33094544

[159] Yu M, Gai C, Li Z Targeted exosome-encapsulated erastin induced ferroptosis in triple negative breast cancer cells. Cancer Sci. 110(10), 3173 (2019). 31464035 10.1111/cas.14181 PMC6778638

[160] Schindler C, Collinson A, Matthews C Exosomal delivery of doxorubicin enables rapid cell entry and enhanced *in vitro* potency. PLoS ONE 14(3), e0214545 (2019). 30925190 10.1371/journal.pone.0214545 PMC6440694

[161] Alvarez-Erviti L, Seow Y, Yin H, Betts C, Lakhal S, Wood MJ. Delivery of siRNA to the mouse brain by systemic injection of targeted exosomes. Nat. Biotechnol. 29(4), 341–345 (2011). 21423189 10.1038/nbt.1807

[162] Ohno SI, Takanashi M, Sudo K Systemically injected exosomes targeted to EGFR deliver antitumor microRNA to breast cancer cells. Mol. Ther. 21(1), 185–191 (2013). 23032975 10.1038/mt.2012.180 PMC3538304

[163] Tian Y, Li S, Song J A doxorubicin delivery platform using engineered natural membrane vesicle exosomes for targeted tumor therapy. Biomaterials 35(7), 2383–2390 (2014). 24345736 10.1016/j.biomaterials.2013.11.083

[164] Fitts CA, Ji N, Li Y, Tan C. Exploiting exosomes in cancer liquid biopsies and drug delivery. Adv. Healthc. Mater. 8(6), 1801268 (2019). 10.1002/adhm.201801268 30663276

[165] Barile L, Vassalli G. Exosomes: therapy delivery tools and biomarkers of diseases. Pharmacol. Ther. 174, 63–78 (2017). 28202367 10.1016/j.pharmthera.2017.02.020

[166] Van Woensel M, Wauthoz N, Rosière R Development of siRNA-loaded chitosan nanoparticles targeting galectin-1 for the treatment of glioblastoma multiforme via intranasal administration. J. Control. Rel. 227, 71–81 (2016). 10.1016/j.jconrel.2016.02.032 26902800

[167] Liao W, Du Y, Zhang C Exosomes: the next generation of endogenous nanomaterials for advanced drug delivery and therapy. Acta Biomater. 86, 1–14 (2019). 30597259 10.1016/j.actbio.2018.12.045

[168] Cruz-Rodriguez L, Dilsiz N, Barea R The algorithms Cruz Rodriguez (CR) are proposing a novel vaccine RNA-peptide against breast, ovarian, and lung cancers disease: exosomes as carrier in cancer progression and metastasis. J. Med.- Clin. Res. Rev. 5(2), 1–16 (2021).

[169] Bu H, He D, He X Exosomes isolation analysis and applications in cancer detection and therapy. Chembiochem. 20, 451–461 (2019). 30371016 10.1002/cbic.201800470

[170] Aqil F, Munagala R, Jeyabalan J, Agrawal AK, Gupta R. Exosomes for the enhanced tissue bioavailability and efficacy of curcumin. AAPS J. 19(6), 1691–1702 (2017). 29047044 10.1208/s12248-017-0154-9

[171] Illes B, Hirschle P, Barnert S, Cauda V, Wuttke S, Engelke H. Exosome-coated metal–organic framework nanoparticles: an efficient drug delivery platform. Chem. Mater. 29(19), 8042–8046 (2017).

[172] Fathi P, Rao L, Chen X. Extracellular vesicle-coated nanoparticles. View 2(2), 20200187 (2021).

[173] Liang Y, Duan L, Lu J, Xia J. Engineering exosomes for targeted drug delivery. Theranostics 11(7), 3183 (2021). 33537081 10.7150/thno.52570 PMC7847680

[174] Ha D, Yang N, Nadithe V. Exosomes as therapeutic drug carriers and delivery vehicles across biological membranes current perspectives and future challenges. Acta Pharm. Sin. B. 6, 287–296 (2016). 27471669 10.1016/j.apsb.2016.02.001 PMC4951582

[175] Datta A, Kim H, McGee L High-throughput screening identified selective inhibitors of exosome biogenesis and secretion: a drug repurposing strategy for advanced cancer. Sci. Rep. 8(1), 1–13 (2018). 29802284 10.1038/s41598-018-26411-7 PMC5970137

[176] Von Schulze A, Deng F. A review on exosome-based cancer therapy. J. Cancer Metastasis Treat. 6, 1–10 (2020).34778566

[177] Tang Z, Li D, Hou S, Zhu X. The cancer exosomes: clinical implications, applications and challenges. Int. J. Cancer 146(11), 2946–2959 (2020). 31671207 10.1002/ijc.32762

